# Bottom-up Fabrication of Graphene on Silicon/Silica Substrate via a Facile Soft-hard Template Approach

**DOI:** 10.1038/srep13480

**Published:** 2015-08-27

**Authors:** Yuxing Yang, Ruili Liu, Jiayang Wu, Xinhong Jiang, Pan Cao, Xiaofeng Hu, Ting Pan, Ciyuan Qiu, Junyi Yang, Yinglin Song, Dongqing Wu, Yikai Su

**Affiliations:** 1 State Key Laboratory of Advanced Optical Communication Systems and Networks, Department of Electronic Engineering, Shanghai Jiao Tong University, Shanghai 200240, China; 2 School of Physical Science and Technology, Soochow University, Soochow 215006, China; 3 School of Chemistry and Chemical Engineering, Shanghai Jiao Tong University, Shanghai, 200240, China

## Abstract

In this work, a novel soft-hard template method towards the direct fabrication of graphene films on silicon/silica substrate is developed via a tri-constituent self-assembly route. Using cetyl trimethyl ammonium bromide (CTAB) as a soft template, silica (SiO_2_) from tetramethoxysilane as a hard template, and pyrene as a carbon source, the self-assembly process allows the formation of a sandwich-like SiO_2_/CTAB/pyrene composite, which can be further converted to high quantity graphene films with a thickness of ~1 nm and a size of over 5 μm by thermal treatment. The morphology and thickness of the graphene films can be effectively controlled through the adjustment of the ratio of pyrene to CTAB. Furthermore, a high nonlinear refractive index *n*_2_ of ~10^−12^ m^2^ W^−1^ is measured from graphene/silica hybrid film, which is six orders of magnitude larger than that of silicon and comparable to the graphene from chemical vapor deposition process.

In the last decade, the fascinating properties of graphene such as high electronic conductivity^1^, good optical transmittance^2^, large optical nonlinearity^3^,^4^, and excellent mechanical flexibility^5^ make it an attractive material for next-generation electronic/photonic devices^6^,^7^. Presently, the synthesis of graphene on metal surface by chemical vapor deposition (CVD) process is a major strategy to construct large-area graphene films due to its facility and controllability^8^,^9^. However, complicated post-growth techniques to remove the metal substrates are required for the fabrication of photonic or electronic devices such as transistors^10^, detectors^11^, and modulators^12^ with graphene on a dielectric substrate. The transfer of graphene on insulating or semiconducting substrate, such as Si, SiO_2_, and polyethylene terephthalate (PET), would inevitably induce defects like contamination, wrinkles, and cracks in the graphene film^12^. So far, direct synthesis of graphene film on the surface of arbitrary substrates is still highly challenging for material scientists^13^,^14^,^15^,^16^.

Composed of multiple aromatic rings, polycyclic aromatic hydrocarbons (PAHs) can be viewed as nano-scaled fragments of graphene since they all contain a two-dimensional (2D) sp^2^ carbon framework^17^,^18^. Benefiting from the conjugated aromatic cores, PAHs have a strong tendency to form ordered superstructures and the thermal treatment of these aggregates provides an opportunity to achieve graphitic carbon materials^19^,^20^,^21^. More importantly, the composition, graphitization degree, and physical properties of these materials can be easily modified by the selection of thermal treatment conditions and precursors with different structures^19^,^20^,^21^,^22^. Inspired by these results, it is envisaged that direct growth of graphene on an insulting surface is experimentally feasible by the thermal treatment of a pre-organized PAH film. Herein, we report for the first time a transport-free fabrication of graphene films on Si/SiO_2_ substrate via a soft-hard template method with pyrene, a typical PAH molecule, as the carbon source. In our method, the tri-constituent assembly of pyrene, cetyl trimethyl ammonium bromide (CTAB), and tetramethoxysilane (TMOS) allow the formation of a sandwich-like SiO_2_/CTAB/pyrene composite within the micelles of CTAB as a soft template and layered SiO_2_ as a hard template. The sandwich-like films can be further converted to high quantity graphene film with a thickness of ~1 nm and a size of over 5 μm by thermal treatment. Such bottom-up fabrication of graphene film can be operated on any high thermal resistance substrates and the morphology of the synthesized graphene film can be easily adjusted by changing the added amount of PAH precursor. Benefiting from the transparent SiO_2_ layer, the graphene film within SiO_2_ shells possesses a high optical transparency (92.2 ~ 93.3%) in the visible light range and large nonlinear refractive index *n*_2_ of ~10^−12^ m^2^ W^−1^, which is six orders of magnitude larger than that of silicon^23^ and comparable to that of graphene derived from CVD process^24^, indicating potential applications in graphene based nonlinear optics.

## Results and Discussion

**Figure 1:**
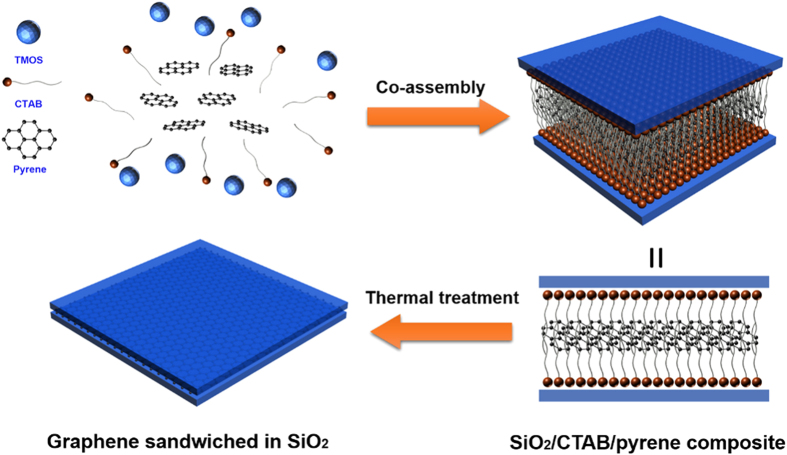
Processing diagram for synthesis of graphene film by the soft-hard template approach.

The overall synthesis procedures of the graphene film are illustrated in Fig. 1. Firstly, the mixture of TMOS and H_2_O was stirred to allow the partially hydrolyzation of TMOS. Then an aqueous solution containing CTAB and pyrene was added^25^. After stirred for a few minutes, the homogeneous solution was spin-coated on a quartz or silicon substrate and dried in air. During this process, the hydrophobic pyrene cannot stay stably in the aqueous solution and tended to go into the CTAB micelles as the soft template. On the other hand, the non-covalent interactions such as ionic forces and hydrogen bonding between the charged part of CTAB and newly synthesized SiO_2_ enable the formation of layers of SiO_2_ on both sides of the CTAB micelles as hard template. As a result, the tri-constituent self-assembly of pre-hydrolyzed TMOS, CTAB, and pyrene produced a sandwich-like SiO_2_/CTAB/pyrene composite^25^. Spin-coating the solution on a Si/SiO_2_ or quartz substrate and thermal treatment of the resulting film at 900 °C in nitrogen flow lead to the formation of graphene films sandwiched in SiO_2_ shells. The molar ratio of TMOS to CATB is fixed as 4:1 and the ratio of pyrene to CTAB is varied from 0:20, 1:20, 2:20, and 3:20. The self-assembled samples before thermal treatment are denoted as PY-0, PY-1, PY-2, and PY-3, respectively. The derived composite films with graphene sandwiched in SiO_2_ are named as GS-0, GS-1, GS-2, and GS-3, respectively. The key aspect of our method is simultaneous employment of surfactant CTAB as a soft template and layered SiO_2_ as a hard template. The micelles of CTAB help the hydrophobic pyrene to form ordered superstructures in aqueous solution. The SiO_2_ layers provide a flat confinement space to prevent the sublimation or aggregation of pyrene and ensure the formation of a thin graphene layer during the thermal treatment. Moreover, for the application of the produced graphene film in optical devices, the SiO_2_ shell can further protect the film from scratching or folding. Etching away the SiO_2_ component with diluted hydrofluoric acid (HF) can release the graphene sheet for structural characterization.

**Figure 2:**
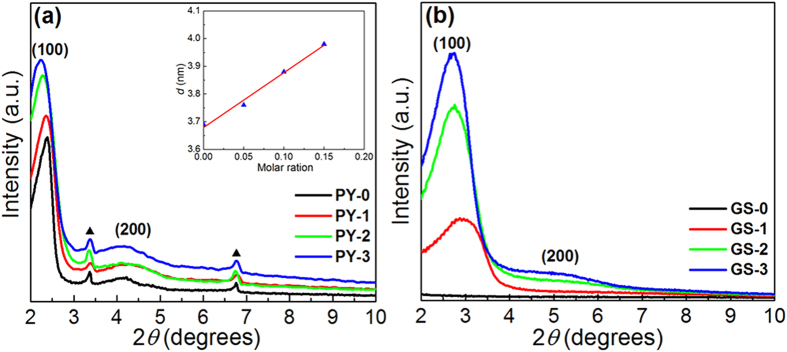
(**a**) XRD patterns of sandwich-like SiO_2_/CTAB/pyrene composites, (**b**) XRD patterns of sandwich-like SiO2/CTAB/pyrene composites after thermal treatment at 900 °C under nitrogen flow. Inset: the variation of the *d* values of SiO_2_/CTAB/pyrene composites as a function of molar ratio of PY to CTAB. The red line shows a linear fit to the experimental data.

The microstructures of the obtained samples are firstly explored with powder small-angle X-ray diffraction (XRD). In the XRD patterns of PY-0, PY-1, PY-2, and PY-3 (Fig. 2a), these pyrene-containing silica-CTAB films manifest characteristic diffractions for the lamellar structures with *d* values of 3.69, 3.76, 3.88, and 3.98 nm, respectively, which exhibit a good linear relationship with the added amount of pyrene. Therefore, the diffractions should correspond to the distances between two neighboring silica layers in the sandwich-like SiO_2_/CTAB/pyrene composite^26^,^27^. On the other hand, a second-order reflection with *d* value of ca. 2.6 nm can also be found in all the XRD spectra of the four samples. The variation of the amount of pyrene shows no influence on the 2*θ* angles of the second-order reflection and the diffractions totally disappear in the XRD spectra of GS-0, GS-1, GS-2 and GS-3, which is thus supposed to be derived from the crystalized CTAB^26^,^28^. After the thermal treatment, no diffraction peaks can be observed in the XRD spectra of GS-0 (Fig. 2b), indicative of the absence of ordered architectures. In contrast, GS-1, GS-2 and GS-3 still retain strong diffractions locating at 2.96°, 2.74° and 2.70°, respectively. Moreover, in the XRD spectra of GS-2 and GS-3, the broad peaks around 5.20° can be assigned to the (200) reflections of a lamellar packing, suggesting these samples still have layered structures. The *d*-spacings for the (100) reflections of GS-1, GS-2 and GS-3 are calculated as 2.98, 3.17 and 3.27 nm, respectively. Compared with the corresponding samples before thermal treatment, the reduced *d* values of GS-1, GS-2 and GS-3 should be owing to the shrinkage of the lamellar architectures in the composites.

**Figure 3:**
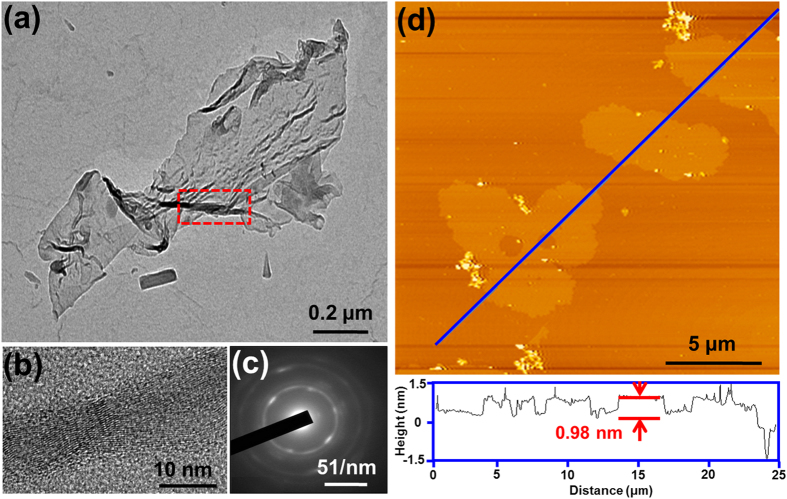
(**a**) TEM image, (**b**) HRTEM image, (**c**) SAED image, (**d**) AFM image of graphene from GS-2 after removal of the silica.

In the Fourier transform infrared (FTIR) spectra of PY-2 (Fig. S1), the strong peaks located at 2922 and 2852 cm^−1^ can be attributed to the C-H stretching from CTAB and pyrene. In contrast, these peaks are absent in the FTIR spectra of GS-2, indicative of the decomposition of surfactant CTAB and the conversion of pyrene to graphene film after the thermal treatment. Additionally, no peaks from silica can be observed in the FTIR spectra of the graphene after the etching process with HF (Fig. S1c), confirming the complete removal of silica. As shown in the scanning electron microscopy (SEM) images of GS-2 (Fig. S2), the cross section of the composite film manifests an obvious layered structure. After the removal of SiO_2_ components in the composite films with HF, the resultant graphene sheets are further investigated by transmission electron microscopy (TEM) and atomic force microscopy (AFM). It is found that the morphology of the graphene sheets show evident dependence on the added amount of pyrene (Fig. 3 and S3). The graphene sheets from GS-1 are irregular discs with diameters of ~100–150 nm. In contrast, the TEM image of the graphene films from GS-2 indicates that they have typical 2D sheet-like morphology with diameters larger than 5 μm. The wrinkles and folds of graphene should be attributed to the absence of the protection from SiO_2_ layers. The high-resolution TEM (HRTEM) result of the wrinkled part of this graphene film shows well-resolved lattice structure with a lattice space of ~0.34 nm, which can be assigned to the (002) plane of graphene^29^. The selected-area electron diffraction (SAED, Fig. 2c) of graphene from GS-2 exhibits a resolved six-fold-symmetry diffraction pattern, similar to that of pristine graphene^30^, indicating that the graphene obtained from our method has a highly crystallized structure. With further increased molar ratio of pyrene to CTAB, the graphene from GS-3 has a diameter even over 10 μm. However, the strong and uneven contrast observed in its TEM image implies that the graphene film is thick and does not have a smooth surface. Moreover, the added amount of pyrene also has an influence on the thickness of derived graphene sheets. As indicated by the AFM image (Fig. S4), the graphene discs from GS-1 have a thickness of around 0.34 nm, which is typical thickness for one layer graphene. While the thickness of the graphene sheets from GS-2 is ~0.97 nm (Fig. 3d), suggesting that they contain 2 ~ 3 layers of graphene. In accordance with the observation in TEM images, the graphene film from GS-3 has a thickness of around 4.39 nm, which is more than 10 times over the thickness of the graphene discs from GS-1.

**Figure 4:**
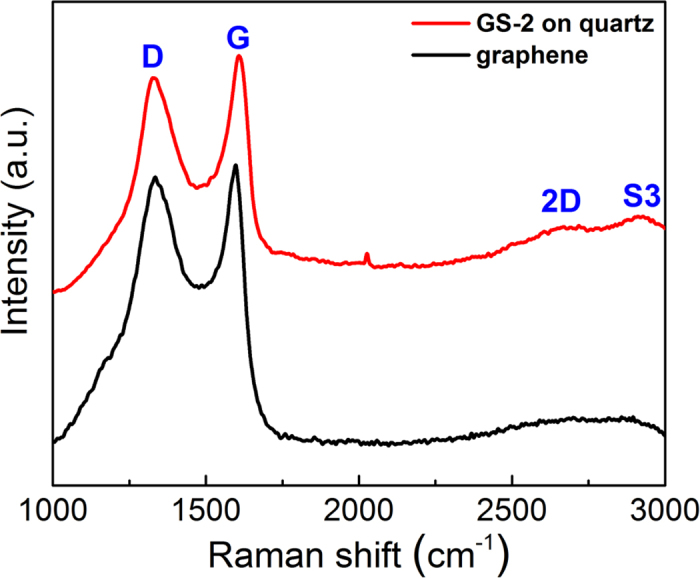
Raman spectra of GS-2 on quartz (red) and the graphene from GS-2 after the removal of silica (black).

To evaluate the quality and uniformity of the graphene films, Raman spectra of GS-2 on quartz and the graphene after the removal of silica were further recorded in this work. As shown in Fig. 4, two major peaks at 1350 and 1580 cm^−1^ can be observed for both GS-2 and the graphene from GS-2 after the removal of silica, which correspond to the D and G bands of carbon, respectively^31^,^32^. The intensity ratio of the D and G band (I_D_/I_G_) of GS-2 and the graphene derived from it are 0.95 and 0.96, respectively, implying both samples have very similar graphitization degrees. Additionally, the 2D peak at ~2700 cm^−1^ and S3 peak at ~2910 cm^−1^ of GS-2 are absent in the Raman spectra of the graphene, which might be due to the wrinkled domains and defects in the graphene without the protection of silica^33^.

**Figure 5:**
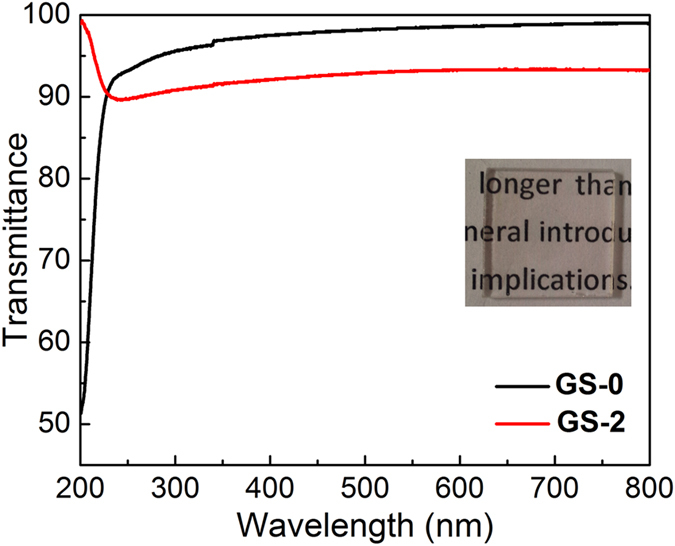
Transmittance of the GS-0 and GS-2 on quartz. The inset is a photograph of GS-2 on quartz.

The easy fabrication of graphene on quartz or Si/SiO_2_ substrate and the unique sandwich-like structure of GS-2 enable it to be used in optical devices. Moreover, as shown in optical microscopy image (Fig. S6), the GS-2 film can be deposited on the surface of quartz with only a few observable defects, indicating its good uniformity and continuity. Inspired by the above results, the optical properties of GS-2 on quartz are investigated by ultraviolet-visible spectrometer and Z-scan measurement. In the visible light region, the transmittance of GS-2 is 92.2 ~ 93.3% (Fig. 5), similar to the previously reported graphene films^34^,^35^. In contrast, the transmittance of pure SiO_2_ film (GS-0) is about 98%. Since the absorbance of an individual layer of graphene is 2.3%^2^, the composite film in GS-2 should contain 2 ~ 3 layers of graphene, which agrees with the results from AFM characterization.

**Figure 6:**
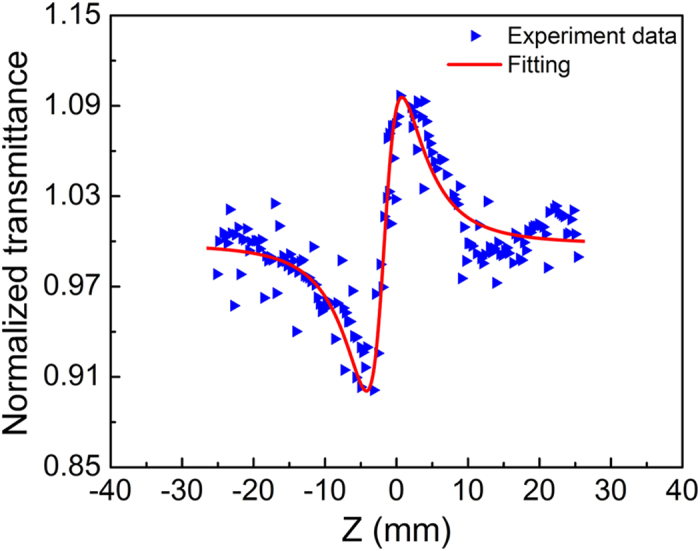
Z-scan trace for GS-2 film on quartz. The red line shows the fitting result.

As an important nonlinear optical parameter, the nonlinear refraction index (*n*_2_)^36^ of GS-2 on quartz is studied by Z-scan measurement. The typical shape of a Z-scan curve with a prefocal transmittance minimum (valley) followed by a postfocal transmittance maximum (peak) can be clearly seen in Fig. 6, indicating that the GS-2 has a positive nonlinear refraction index^37^. In contrast, the transmittance of GS-0 only has small fluctuation along the Z axis, implying that the SiO_2_ component in GS-2 has little influence on the Z-scan measurement results (Fig. S7). As a result, *n*_2_ of GS-2 is calculated to be 10^−12^ m^2^ W^−1^ (Supplementary Information), which is comparable to that of the previously reported graphene films fabricated by CVD method^24^ and six orders of magnitude larger than that of silicon^23^.

In summary, we have developed a facile bottom-up strategy for the direct preparation of graphene on Si/SiO_2_ substrate by using a soft-hard template approach. Sandwiched in SiO_2_ shells, the resulting graphene film shows tunable morphologies via the modification of the molar ratio of the precursors. Formed on a quartz substrate, the graphene films exhibit a high transmittance of 92.2 ~ 93.3% and a large nonlinear coefficient *n*_2_ of ~10^−12^ m^2^ W^−1^, which is comparable to those of CVD graphene films. This synthetic method provides a simple yet efficient route to fabricate graphene films on insulating substrates for optical and electronic applications. More importantly, the adjustable morphology of graphene films via the precursors allows one to deliberately tune the device performance for versatile purposes.

## Methods

In a typical synthesis process of graphene on quartz/silicon, TMOS (1.9 g) was partially hydrolyzed by a substoichiometric amount of water (0.45 mL) under acidic conditions (pH = 3) for 2 hours at room temperature. Then an aqueous solution containing CTAB (1.14 g) and pyrene was added. The molar ratio of pyrene to CTAB varied from 0:20, 1:20, 2:20, and 3:20. After the mixture was stirred for a few minutes, the homogeneous sol was spin-coating on the quartz and silicon substrates. Subsequently, the films were put in fume cupboard until the total vaporization of the solvent. A heat process (100 °C, 24 hours) was then required for the further solidification of the composites. Subsequently, thermal treatment of the silica/CTAB/pyrene composites in a nitrogen flow at 900 °C for 2 hours.

To obtain graphene suspension without silica, the sol above mentioned was transferred into dishes. After the heat treatment at 100 °C for 24 hours, the as-made products were scraped from the dishes and ground into fine powders. Thermal trearment was carried out at 900 °C for 2 hours in a nitrogen flow to get the SiO_2_/graphene/SiO_2_ nanocomposites. The silica frameworks were removed by treating the samples with aqueous HF (10%) for 48 hours.

## Additional Information

**How to cite this article**: Yang, Y. *et al.* Bottom-up Fabrication of Graphene on Silicon/Silica Substrate via a Facile Soft-hard Template Approach. *Sci. Rep.* **5**, 13480; doi: 10.1038/srep13480 (2015).

## Supplementary Material

Supplementary Information
