# Supplementary material for: Bottom-up Fabrication of Graphene on Silicon/Silica Substrate via a Facile Soft-hard Template Approach
Source: Sci Rep. 2015 Aug 27;5:13480. doi: 10.1038/srep13480 (PMC4550833; doi:10.1038/srep13480)
Supplement: Supplementary Information [file srep13480-s1.pdf]

# Supplementary Information for

Bottom-up Fabrication of Graphene on Silicon/Silica Substrate via a Facile Soft-hard Template Approach

*Yuxing Yang[1], Ruili Liu[1]*, Jiayang Wu[1], Xinghong Jiang[1], Pan Cao[1], Xiaofeng Hu[1], Ting Pan[1], Ciyuan Qiu[1], Junyi Yang[2], Yinglin Song[2], Dongqing Wu[3]* & Yikai Su[1]*

[1]*State Key Laboratory of Advanced Optical Communication Systems and Networks, Department of Electronic Engineering, Shanghai Jiao Tong University, Shanghai 200240, China*

[2]*School of Physical Science and Technology, Soochow University, Soochow 215006, China*

[3]*School of Chemistry and Chemical Engineering, Shanghai Jiao Tong University, Shanghai, 200240, China*

**Correspondence to ruililiu@sjtu.edu.cn; wudongqing@sjtu.edu.cn*

## Experimental

All the chemicals are purchased from Sinopharm chemical reagent Co., Ltd and used without further purification.

In a typical synthesis process, tetramethoxysilane (TMOS, 1.9 g) was partially hydrolyzed by a substoichiometric amount of water (0.45 mL, the molar ratio of TMOS: $H_2O$ was 1:2) under acidic conditions (pH = 3) for 2 hours at room temperature. Then an aqueous solution of cetyltrimethylammonium bromide (CTAB, 1.14 g) and pyrene was added. The molar ratio of pyrene to CTAB varied from 0:20, 1:20, 2:20, and 3:20. After the mixture was stirred for a few minutes, the homogeneous sol was coated on the petri dishes. Subsequently, the films were put in fume cupboard until the total vaporization of the solvent. A heat process (100 °C, 24 hours) was then required for the further solidification of the composites. According to the ratio of pyrene to CTAB, the as-made silica/CTAB/pyrene composites were denoted as PY-0 (0:20), PY-1 (1:20), PY-2 (2:20), and PY-3 (3:20), respectively. The subsequent thermal treatment of the silica/CTAB/pyrene composites in a nitrogen flow at 900 °C for 2 hours and the products were named as GS-0 (527 mg), GS-1 (560 mg), GS-2 (550 mg), and GS-3 (564 mg). According to the result of thermal gravimetric analysis (TGA, Fig. S5), the weight content of graphene in GS-1, GS-2, and GS-3 is 2.8 %, 3.0 % and 3.1 %,

respectively. Base on the mass of pyrene, the yield of graphene in GS-1, GS-2, and GS-3 is ca. 49.7 %, 26.2 %, and 18.5 %, respectively.

To obtain graphene without silica, the silica frameworks were removed using aqueous HF (10 %), and then dialyzed thoroughly with Millipore water.

For the preparation of graphene/silica hybrid films on silicon and quartz, the above obtained sol (0.1 mL) was spin-coated on the substrate with the size of 1.5 cm x 1.5 cm for 1 minute with a coating spin speed of 3000 rpm. A heat process (100 °C, 24 hours) and then thermal treatment of the resulting films in a nitrogen flow at 900 °C for 2 hours led to the hybrid film on corresponding substrates.

**Characterization:**

Small-angle X-ray diffraction (XRD) patterns were obtained by a X-Ray Polycrystaline diffractometer (D8 DA VINCE, Bruker, Germany) equipped with Cu-K$\alpha$ radiation of 1.54 Å. Fourier transform infrared (FTIR) spectra were recorded the function group in the sample using Fourier transform infrared spectrometer (AVATVR370, Nicolet, America). A field emission scanning electron microscope (FESEM, Ultra Plus, Zeiss, Germany) was used to observe the lamellar structure of the sample. Simultaneous DSC-TGA (Q600, TA, America) was employed to detect the change of mass during the thermal treatment process. Before calcination, the samples were dried for 3 hours in the oven at 70 ºC. The morphology and crystal structure were studied by high-resolution transmission electron microscope (HRTEM, JEM-2010F, JEOL). The samples for the TEM tests were prepared by the evaporation of one drop of the solution onto a lacey support film. An acceleration voltage of 200 kV was used throughout the measurements. The thickness of graphene was identified by atomic force microscopy (AFM, Multimode Nanoscope III, Bruker, Germany). The samples for AFM measurement were prepared by spotted onto freshly cleaved mica surface and dried in air (~20 minutes). The samples were imaged in air by tapping mode under ambient conditions.

Dispersive Raman microscope (Senterra R200-L, Bruker Optics, Germany), ultraviolet-visible (UV/Vis) spectrophotometer (J-4100, Hitachi, Japan) and Z-scan

(School of Physical Science and Technology, Soochow University) measurements were used to investigate the properties of graphene/silica hybrid film on quarz. The excitation wavelength of Raman spectra is 532 nm at room temperature. The Z-scan measurement setup has been used as previously reported[S1].

The sample is subjected to pulses emitted form a Nd:YAG picoseconds laser with center wavelength 532 nm, pulse duration 21 ps (full width at half-maximum, FWHM), and repetition rate 10 Hz. The two regimes: open aperture and closed aperture are performed in our experiment. The sensitivity to nonlinear refraction is entirely due to the aperture and removal of the aperture completely eliminates the effect[S2]. Therefore, nonlinear absorption coefficients can be extracted from open-aperture experiments at first. Then closed-aperture measurement was carried out. Upon dividing by the open-aperture curve, the nonlinear refractive index was obtained. The up-down curve suggests that a small positive on-axis phase shift $\Delta\Phi$ (i.e. self-focusing occurs). The magnitude of the phase shift can be determined from the change in normalized transmittance between peak and valley. In the limit of small nonlinear phase change and the far-field condition $d >> z_0$, the curve can be fitted by equation (1):

$$T(x) = 1 + \frac{4x\Delta\emptyset}{(1+x^2)(9+x^2)} \qquad (1)$$

where $x=z/z_0$ is the normalized distance from the focus and $z_0$ is the Rayleigh length.

The nonlinear refractive index $n_2$ can be calculated using the equation (2):

$$n_2 = \Delta\Phi/(k_0LI) \qquad (2)$$

where $k_0 = 2\pi/\lambda$ , $L$ is the sample thickness and $I$ is the peak intensity of the laser.[S3]

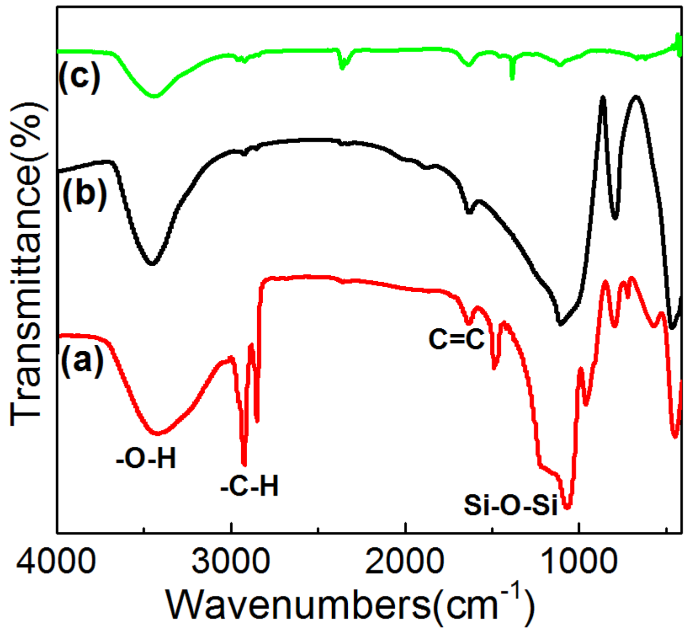


Figure S1. FT-IR spectra of (a) PY-2,(b) GS-2, and (c) the graphene obtained from GS-2 after removal silica component.

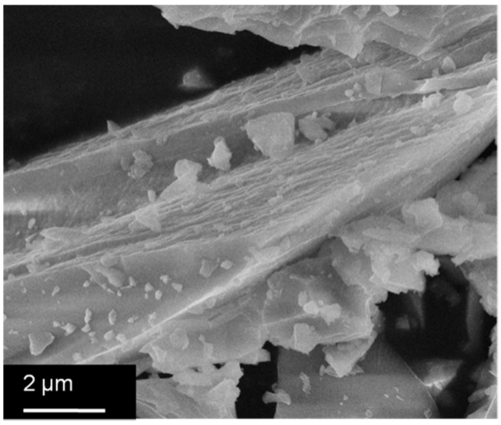


Figure S2. SEM images of GS-2 film after thermal treatment at 900 °C.

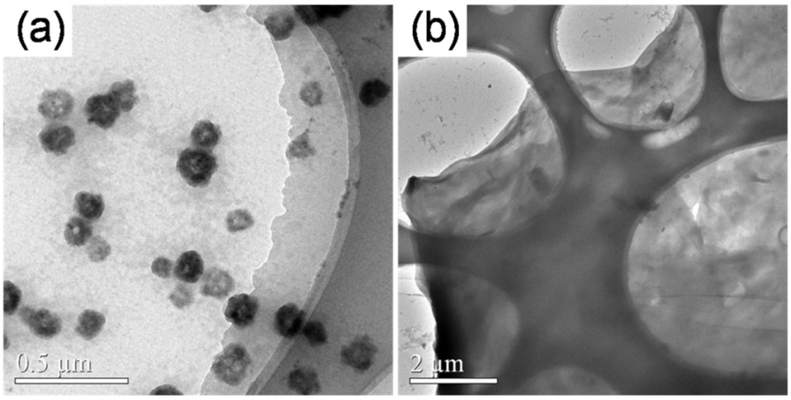


Figure S3. TEM images of graphene sheets (a) derived from GS-1 and (b) derived from GS-3 after removing the $SiO_2$ component.

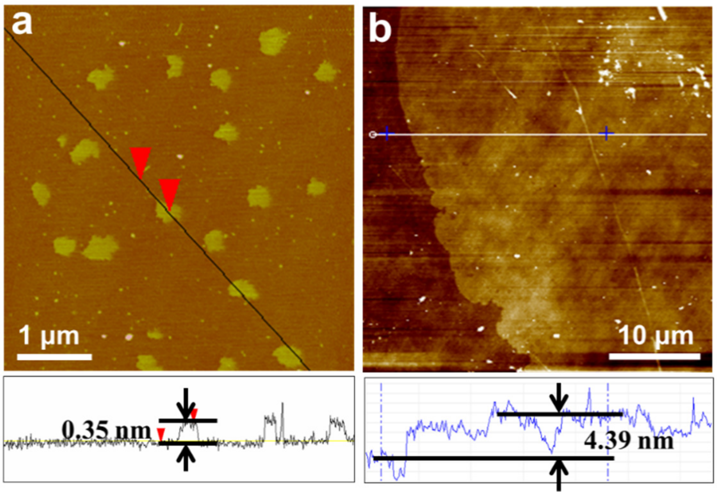


Figure S4. AFM images of graphene films (a) derived from GS-1 and (b) derived from GS-3 after removing the $SiO_2$ component.

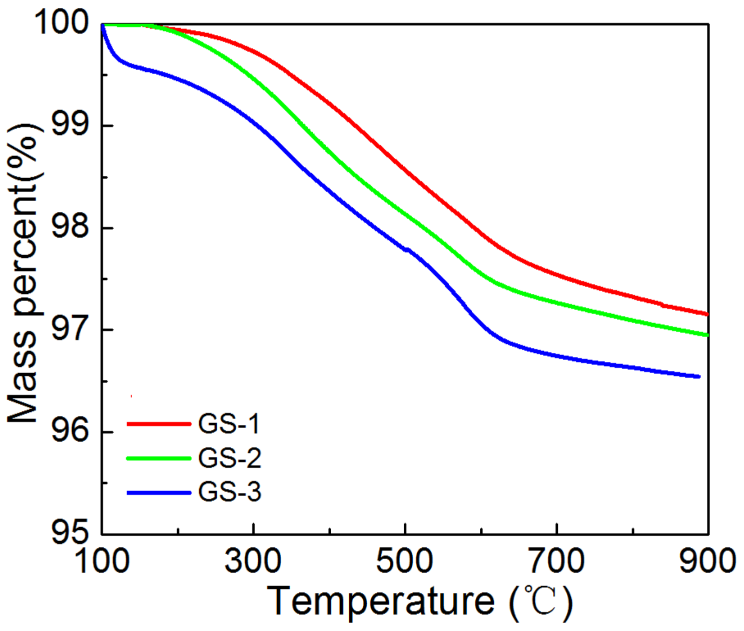


Figure S5. Thermal gravimetric analysis of GS-1, GS-2, and GS-3 at 900 $^{o}$C under air flow, the heating rate is 10 $^{o}$C/min.

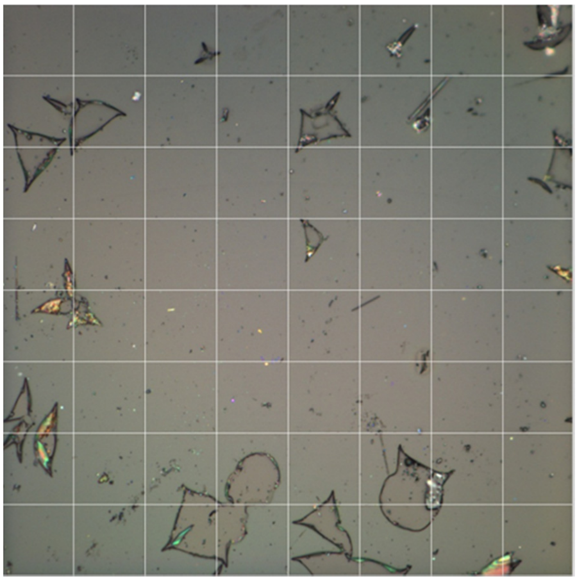

Figure S6. Optical microscope image of GS-2 on quartz. Every grid represent 50 micrometers.

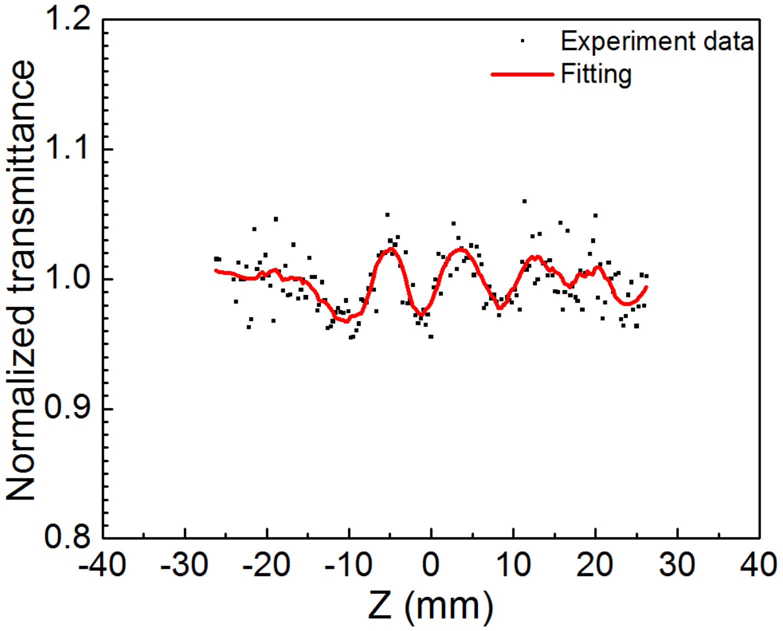


Figure S7. Z-scan trace for GS-0 on quartz. The red line shows the fitting result.

**References:**


S1. Moreels, I., Hens, Z., Kockaert, P., Loicq, J. & Thourhout, D. V. Spectroscopy of the nonlinear refractive index of colloidal PbSe nanocrystals, *Appl. Phys. Lett.* **89**, 193106 (2006).

S2. Mansoor, S. B., Wei, T. H., Hagan, D. J. & Van Stryland, E. W. Sensitive measurement of optical nonlinearities using a single beam. *IEEE J. Quantum Elect.* **26**, 760-769 (1990).

S3. Zhang, H. Z-scan measurement of the nonlinear refractive index of graphene. *Opt. Lett.,* **37***,* 1856-1858 (2012).
